# Prevalence of urinary abnormalities in asymptomatic school going children by dipstick method

**DOI:** 10.12669/pjms.40.11.8878

**Published:** 2024-12

**Authors:** Hassan Nawaz, Noman Butt, Muhammad Anees, Kainat Babar

**Affiliations:** 1 Hassan Nawaz, MBBS. Department of Nephrology, King Edward Medical University, Lahore, Pakistan; 2 Noman Butt, FCPS. Department of Nephrology, King Edward Medical University, Lahore, Pakistan; 3 Muhammad Anees, FCPS. Department of Nephrology, King Edward Medical University, Lahore, Pakistan; 4 Kainat Babar, BSc. RDT. Department of Nephrology, King Edward Medical University, Lahore, Pakistan

**Keywords:** Urinary abnormalities, Screening methods, Dipstick testing, Urinalysis, Renal disorders

## Abstract

**Objective:**

To determine the prevalence of urinary abnormalities in asymptomatic school children.

**Methods:**

This observational study was conducted at King Edward Medical University/Mayo Hospital Lahore. Children of 5-15 year of age from Central Model School, Lower Mall, Lahore were included from March to May, 2022. Children having prior renal or systemic disease, on steroids and menstruating girls at the time of urinalysis were excluded. In first screening, children’s urine was collected in clean 20mL vessel and examined using urinary dipstick. Children with aberrant results were re-screened after two weeks.

**Result:**

Out of total 1600 children, majority were male 1416(88.5%) with mean age 11.92±2.47 years. Urinary abnormalities were observed in 278(17.38%) children on first screening and 86(5.38%) on second screening. Isolated proteinuria was present in 131(8.19%) children on first screening and 26(1.62%) on second screening. Isolated hematuria was present in 100(6.50%) on first screening and 33(2.06%) on second screening. Isolated pyuria was present in 24(1.50%) on first screening and 14(0.88%) on second screening. Combined proteinuria and hematuria were present in 10(0.62%) on first screening and two (0.12%) on second screening. Combined proteinuria and pyuria were present in three (0.19%) on first screening and three (0.19%) on second screening. Combined hematuria and pyuria were present in nine (0.56%) on first screening and five (0.31%) on second screening. Combined proteinuria, hematuria and pyuria was present in one (0.06%) on first screening and three (0.19%) on second screening.

**Conclusion:**

Urine screening is necessary for early detection and management of kidney diseases. Marked number of children was found having urinary abnormalities. Asymptomatic proteinuria was the most common finding reflecting underlying kidney pathology going undiagnosed.

## INTRODUCTION

Chronic Kidney disease is one of the leading causes of death. CKD is not only the problem of the developed countries but is also affecting countries with lower middle income. According to systematic review conducted in 2017, worldwide burden of CKD was 843.6million.^1^ CKD affects multiple organs of body along with social life, loss of productivity and financial burden.^2^ For early detection of kidney disease, National Kidney foundation (NKF) started Kidney Early Evaluation Program (KEEP). Urine analysis is one of the important screening tools for early detection of kidney diseases due to low cost.^3^ In some countries annual screening program of school children is done regularly like Japan, Taiwan, Nigeria and South Korea.^4^ Urine dipstick test helps in detection of asymptomatic hematuria or proteinuria with a prevalence reported between 1.0-10.0% for proteinuria and 0.52-2.0% for hematuria. Notably, most kidney diseases are asymptomatic until the disease has progressed; therefore, mass screening helps determine the prevalence of kidney disease in asymptomatic children.^5^ In Pakistan, there is no data available on this important aspect, so this study was conducted.

## METHODS

This cross sectional study was conducted at Nephrology Department, Mayo Hospital Lahore, in collaboration with Central Model School, Lower Mall, Lahore. Data was collected from March-2022 to May-2022. Written consent was taken from parents of the children.

### Ethical Approval:

Permission was taken from Institutional Review Board of King Edward Medical University Lahore via letter No. 413/RC/KEMU, dated March 29, 2022 and Board Management of concerned school.

### Inclusion criteria:

- Children between ages of 5-15 years.
- Children having no renal symptoms.

### Exclusion criteria:

- Children having prior renal or systemic disease.
- Children on steroids and drugs which can give false positive/false negative dipstick results.
- Menstruating girls at the time of urinalysis.
- Children whose parents did not give consent.

### Data collection and analysis:

For 95% confidence interval with margin of error 0.5% and incidence of hematuria as 1%,^6^ the sample size is as n=1521:


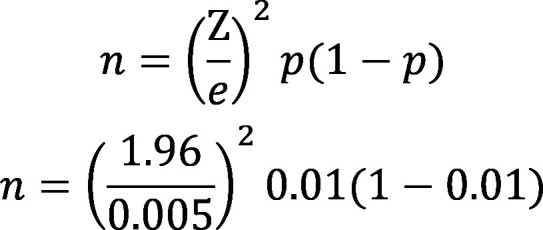


On first screening demographic data (name, age, gender, class) and anthropometric measurements (height, weight, BMI) were noted. Children’s urine was collected in clean 20mL vessel and examined for urinary abnormalities using urinary dipstick (CombiScreen) by immersing it in urine for five seconds and interpreting after one minute. Urine parameters analyzed were specific gravity, protein, blood and leukocytes. Children with aberrant results were recalled after 15 days. Their urine was reanalyzed for protein, blood and leukocytes. Hematuria was defined as having >05 RBCs per high power field and pyuria as having >25 WBCs per high power field. SPSS 25.0 was used for statistical analysis. The categorical variables were expressed as frequencies and percentages. The relative risk was used to find the relationship between predictors and abnormalities. Proportions and mean differences were compared using Chi-square and Student’s t-tests respectively. The *p-value <0.05* was considered significant.

## RESULTS

Sixteen hundred children were included. Majority of the students were male 1416(88.50%), while 184(11.50%) were female. Mean age of the students was 11.92±2.47 years and most of the students 1306(81.62%) were of >10 years. Mean BMI was 18.35+3.92 with range of 10.2-44.4. Most of the children were healthy 985(61.56%) while rest of them were underweight, overweight and obese 27(1.69%), 206(12.88%), 132(8.25%) respectively. Urinary abnormalities were in 278(17.38%) children on first screening and 86(5.38%) on second screening. Isolated proteinuria was present in 131(8.19%) children on first screening [trace 90(68.70%), + 28(21.37%), ++ 11(8.40%), +++ 2(1.53%)] and 26(1.62%) on second screening [trace 19(73.08%), + 5(19.23%), ++ 2(7.69%)].

**Fig.1:**
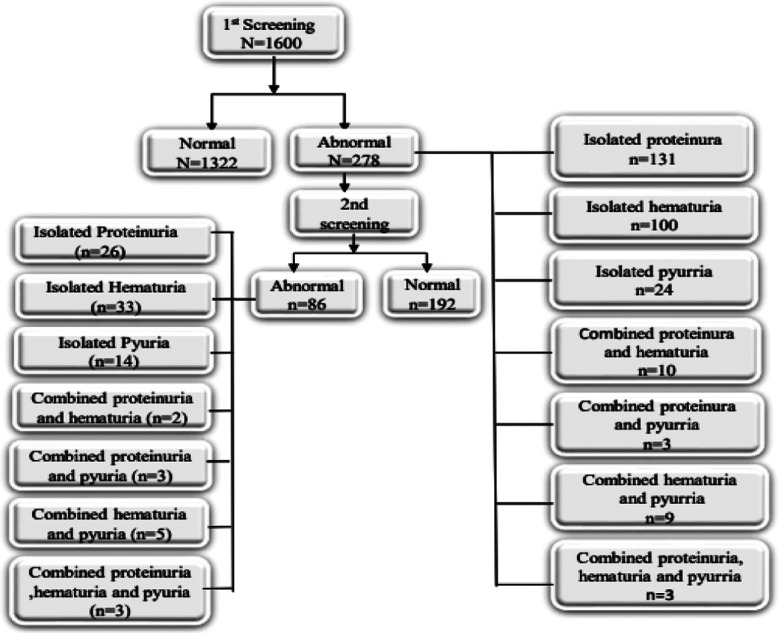
Urinary abnormalities in first screening and second screening.

Isolated hematuria was present in 100(6.50%) children on first screening [5-10RBCs/HPF 85(85.0%), 50RBCs/HPF 13(13.0%), 300RBCs/HPF 2(2.0%)] and 33(2.06%) on second screening [5-10RBCs/HPF 26(78.79%), 50RBCs/HPF 5(15.15%), 300RBCs/HPF 2(6.06%)]. Isolated pyuria was present in 24(1.50%) children on first screening and 14(0.88%) on second screening. Combined proteinuria and hematuria were present in 10(0.62%) children on first screening and two (0.12%) on second screening. Combined proteinuria and pyuria were present in three (0.19%) children both on first and second screening. Combined hematuria and pyuria were present in nine (0.56%) children on first screening and five (0.31%) on second screening. Combined Proteinuria, hematuria and pyuria were present in one (0.06%) child on first screening and three (0.19%) on second screening (Fig.1).

Urinary abnormalities showed significant disparity on gender basis as they were more common in females (*p= 0.003*) because of increased prevalence of isolated hematuria (Male 14% vs. females 25%, *p=0.009*) and isolated pyuria (Male 05% vs. Female 32%, *p<0.001)*. Children having positive family history of renal diseases had more urinary abnormalities (*p<0.001*). Also, prevalence of urinary abnormalities increased with increasing age (*p=0.008*). Urinary abnormalities showed no significant difference on basis of past history of fever within last two weeks (*p=0.601*) and BMI having underweight (*p=0.797*) and overweight (*p=0.485*) (Table-I).

**Table-I:** Differences in urinary abnormalities on basis of gender, age group, family history, past history of fever and BMI.

Variable	Abnormality			RR (95% CI)	P-value

	Present (n=86)	Absent (n=192)			
Gender	Female	20 (23.3%)	21 (10.9%)	1.75(1.20-2.55)	0.003
	Male	66(76.7%)	171(89.1%)		
Age group	Less than 12 years	55 (63.9%)	89 (46.4%)	1.65(1.14-2.40)	0.008
	12 years and more	31 (36.1%)	103(53.6%)		
Family History of kidney disease	Yes	16 (18.6%)	12 (6.25%)	2.04(1.40-2.98)	<0.001
	No	70 (81.4%)	180 (93.75%)		
Past History of Fever	Yes	13 (15.1%)	34(17.7%)	0.88(0.53-1.44)	0.601
	No	73 (84.9%)	158(82.3)		
BMI	Healthy	57 (66.3%)	120(62.5%)	0.94(0.61-1.47)	0.797
	Underweight	17 (19.8%)	39(20.31%)		
	Overweight	12 (13.9%)	33(17.19%)	0.81(0.47-1.37)	0.437

## DISCUSSION

Final results of this study showed quite significant number of children having silent urinary abnormalities. It showed total abnormalities in 5.38%, isolated proteinuria in 1.63%, isolated hematuria in 2.06%, isolated pyuria in 0.88%, proteinuria with hematuria in 0.12%, proteinuria with pyuria in 0.19%, hematuria with pyuria in 0.31% and combined proteinuria with hematuria & pyuria in 0.19% after second screening, (Fig.1).

Diagnosis of renal or urinary tract illness among asymptomatic patients may be delayed. This delay can be avoided by use of dipstick technique, which is the quickest, non-invasive and economical screening method.^7^ Urine dipstick is easy to perform, readily available and does not require much of manpower and infrastructure. Previously, different renal diseases were found on screening of children, among them IgA nephropathy were the most leading cause.^8^,^9^ In developed countries, congenital diseases account for majority of cases of CKD in children. However, infections and acquired diseases are the leading causes of CKD in children in developing countries, who are diagnosed at much later stages, possibly due to lack of resources and screening programs.^10^Also increase in prevalence of obesity and hypertension in pediatric population is causing increase in pediatric CKD patients, which is ultimately causing more children on renal replacement therapy.^11^

The incidence of ESRD in children ranges from 5-6/million children under the age of 15 years in Europe, Australia, and Japan; and 10-11/million children inUSA.^12^ Mortality is quite high in ESRD children than general pediatric population.^13^ Dialysis and transplantation may be avoided or delayed if population-based preventive measures are taken. Lack of financial resources, dialysis clinics, equipment’s, and skilled employees make prevention increasingly vital in our country.^4^

In this study, total urinary abnormalities were found in 86(5.38%) children. This finding was in contrast to studies done in Egypt, Turkey, Jammu and Kashmir (India), Andhra Pradesh (India), Iran and China which showed prevalence of total urinary abnormalities 3.30%, 1.14%, 2.63%, 2.28%, 0.80% and 0.84% respectively,^4^,^14^-^18^(Table-II). These studies showed prevalence of urinary abnormalities much less as compared to our study. The higher prevalence of urinary abnormalities in this study is due to higher number of subjects having isolated proteinuria and isolated hematuria as compared to previous studies.

**Table-II:** Comparison of urinary abnormalities among different countries.

Parameters	Present Study (Pakistan) N=1600	Shalaby et al CITATION Sha19 \l 1033 (13) (Egypt) N=1000	Muradova et al CITATION MUR20 \l 1033 (14) (Turkey) N=1052	Benergee et al CITATION Ban22 \l 1033 (15) (India) N=1675	Srinivasulu et al CITATION Sri \l 1033 (4) (India) N=1626	Nodoshan et al CITATION AAH15 \l 1033 (16) (Iran) N=3014	Jia et al CITATION Jia21 \l 1033 (17) (China) N=11753
Year of study	2022	2019	2020	2022	2018	2015	2015-19
Total Screened	1600	1000	1052	1675	1626	3014	11753
Positive on 1^st^ screen	278(17.38%)	66(6.60%)	137(13.02%)	76(4.54%)	45(2.76%)	94(3.12%)	
Isolated Proteinuria	131(8.19%)	10(1.0%)	22(2.09%)	4(0.24%)	5(0.31%)	54(1.79%)	
Isolated Hematuria	100(6.25%)	27(2.70%)	16(1.52%)	32(1.91%)	14(0.86%)	15(0.50%)	
Combined proteinuria with hematuria	10(0.63%)	3(0.30%)	1(0.10%)		3(0.19%)		
Isolated pyuria	24(1.50%)	25(2.50%)	96(9.13%)	39(2.33%)	22(1.36%)	10(0.33%)	
Positive on last screen	86(5.38%)	33(3.30%)	12(1.14%)	44(2.63%)	37(2.28%)	24(0.80%)	99(0.84%)
Isolated proteinuria	26(1.63%)	4(0.40%)	2(0.19%)	2(0.12%)	3(0.19%)	11(0.36%)	51(0.43%)
Isolated hematuria	33(2.06%)	9(0.90%)	8(0.76%)	13(0.78%)	10(0.62%)	2(0.07%)	42(0.36%)
Combined proteinuria and hematuria	2(0.12%)	1(0.10%)	0		3(0.19%)	2(0.07%)	6(0.05%)
Isolated pyuria	14(0.88%)	18(1.80%)	0	21(1.25%)	20(1.23%)	1(0.03%)	

In the present study, isolated proteinuria was detected in 1.63% of cases. This percentage is higher than mentioned in previous studies i.e. 0.40% in Egypt, 0.19% in Turkey, 0.12% in India, 0.19% again in India, 0.36% in Iran and 0.43% in China, (Table-II).^4^,^14^-^18^ This proteinuria can be due to minimal change disease, focal segmental glomerulonephritis, IgA-nephropathy, post-infectious glomerulonephritis, and mesangioproliferative glomerulonephritis. To diagnose this, further work up is required. This high percentage of children having proteinuria may also be attributed to the non-availability of early morning urine sample, orthostatic/exercise induced proteinuria, high percentage of children having concentrated urine and low specificity of urine dipstick. Proteinuria is thought to be a significant independent risk factor for end-stage renal disease. Hence, asymptomatic proteinuria should be screened for early diagnosis and treatment.

Isolated hematuria was the most common abnormality in our study (2.06%), which is higher than researches conducted in other countries like in Egypt (0.90%), Turkey (0.76%), India (0.78%), India (0.62%), Iran (0.07%) and China (0.36%).^4^,^14^-^18^ (Table-II). This high percentage of hematuria in children may be due to presence of urinary tract infections, stone disease, IgA nephropathy, post-infectious glomerulonephritis or urinary tract neoplasm. Proper history, examination, radiological and laboratory investigations will be helpful for making exact diagnosis.

Our study showed that isolated hematuria was almost as common as isolated proteinuria. Children having isolated pyuria (0.88%) were lesser in number as compared to children having isolated hematuria and isolated proteinuria. Our results were lower than Egypt (1.8%), India (1.25%) and India (1.23%)^14^-^16^ but higher than Iran (0.03%) and Turkey (0%)^15^,^17^ (Table-II). Childhood UTIs are the most common type of infections after respiratory and gastrointestinal infections. These can have severe and various complications including hypertension, chronic kidney disease, scars in renal tissue, and disorders of the urinary tract. Therefore, these infections should be timely diagnosed and treated appropriately.^12^

In our study combined proteinuria and hematuria was found in 0.12%, which was almost similar to the results obtained in Egypt 0.10%, India 0.19%, Iran 0.07% and China 0.05% (TableII).^4^,^14^,^17^,^18^ Cause of combined proteinuria with hematuria may be post-infectious glomerulonephritis, IgA-nephropathy, mesangioproliferative glomerulonephritis, stones or UTI’s.

We noted another finding in this study that concentrated urine was present in 1311(81.9%) of children on the basis of high specific gravity (>1.020) because of decreased water consumption, as children fear to seek permission from their teachers to drink water and try avoiding going to toilets because of poor sanitary conditions. This finding is in contrary to the study done in Pakistan which showed low urinary volume in just 34.3% children.^19^

### Limitations:

In this study non-availability of early morning urine samples was the main limitation. Also, majority of children belong to lower-middle socioeconomic class and study was conducted in urban school whom results may not reflect the entire population. Also long-term follow up of children with urinary abnormalities was not done.

## CONCLUSION

Urine screening is necessary for early detection and management of kidney diseases. Marked number of children was found having urinary abnormalities. Asymptomatic proteinuria was the most common finding reflecting underlying kidney pathology going undiagnosed.

### Recommendations:

The medical, social and economic burden of CKD is growing all across Pakistan because of lack of screening programs to diagnose asymptomatic kidney diseases. There is scarcity of data regarding this aspect. Keeping in view the findings of current study national screening programs can be devised for prevention and early diagnosis of kidney diseases. Children should be encouraged to drink more water and sanitary conditions in toilets should be improved so that incidence of kidney stones and voiding dysfunction can be decreased.

### Authors’ Contribution:

**HN:** Data collection, financed, manuscript writing.

**NB:** Did data collection, Literature search, critical review and manuscript editing.

**MA:** Did statistical analysis, review and final approval of manuscript.

**KB:** Did data collection, manuscript editing.

All authors have read the final version and approved it and are also responsible le for integrity of the study.
